# Native speakers like affixes, L2 speakers like letters? An overt visual priming study investigating the role of orthography in L2 morphological processing

**DOI:** 10.1371/journal.pone.0226482

**Published:** 2019-12-23

**Authors:** Laura Anna Ciaccio, Gunnar Jacob

**Affiliations:** Potsdam Research Institute for Multilingualism, University of Potsdam, Potsdam, Germany; Leiden University, NETHERLANDS

## Abstract

In an overt visual priming experiment, we investigate the role of orthography in native (L1) and non-native (L2) processing of German morphologically complex words. We compare priming effects for inflected and derived morphologically related prime-target pairs versus otherwise matched, purely orthographically related pairs. The results show morphological priming effects in both the L1 and L2 group, with no significant difference between inflection and derivation. However, L2 speakers, but not L1 speakers, also showed significant priming for orthographically related pairs. Our results support the claim that L2 speakers focus more on surface-level information such as orthography during visual word recognition. This can cause orthographic priming effects in morphologically related prime-target pairs, which may conceal L1-L2 differences in morphological processing.

## Introduction

The issue of how morphologically complex words are processed by non-native (L2) speakers of a given language, as compared to native (L1) speakers, has been subject to considerable debate in recent years: While some theoretical accounts of L2 morphological processing [1] suggest that L2 speakers rely relatively less on morphological decomposition and more on storage of whole word-form representations in the mental lexicon, other accounts [2] instead suggest that L1 and L2 speakers rely on the same basic mechanisms, and thus argue against fundamental L1-L2 differences. The debate is further complicated by that fact that, even for L1 speakers, the mechanisms involved in the processing of complex words and the way in which such words are represented in the mental lexicon are not entirely clear.

A key experimental approach employed by a considerable number of studies that have contributed to this debate is *morphological priming*. In such studies, participants are typically confronted with pairs of words sharing the same stem (e.g. *walker-walk*) and have to perform a task (such as lexical decision or naming) on the latter of the two words. If the internal structure of a complex word such as *walker* is accessed during processing, this should cause pre-activation of the stem *walk* while processing the prime word *walker*, which should facilitate subsequent processing of the target word *walk* relatively to a control pair such as *spoon-walk*.

The majority of L2 processing studies based on this paradigm have relied on *masked priming*, in which the prime words are presented only very briefly (typically between 30 and 70 ms) and preceded by a visual mask. This usually prevents conscious recognition of the prime. While masked priming studies have undoubtedly provided detailed insight into the mechanisms underlying L1 and L2 morphological processing, the issue of what exactly causes masked morphological priming effects is still subject to considerable debate. Some accounts suggest that masked priming may tap into an early, pre-lexical stage of visual word recognition [3, 4], which is largely semantically blind and specifically concerned with automatic morpho-orthographic decomposition. Other accounts instead assume a considerably more abstract form of decomposition mechanism, which segments complex words into stems plus morphological features [5]. Yet other accounts argue against decomposition, and instead assume that each complex word possesses its own entry in the mental lexicon [6, 7] with morphological priming effects being due to a combination of similarity in form and meaning [8].

L2 studies employing the masked priming paradigm have come to different conclusions about how and whether L2 morphological processing differs from L1 processing. Some priming studies [9, 10] report L1-L2 differences with regard to morphological priming effects. This result is consistent with the idea that L1 processing relies mainly on morphological decomposition of complex words, while L2 processing is rather based on storage and retrieval of full word forms in the mental lexicon. A substantial number of other studies, however [2, 11–16], found similar morphological priming effects in L1 and L2. While some of these studies still argue that complex words are decomposed, and only reject L1-L2 differences with regard to morphological decomposition [13], others reject the idea of decomposition entirely, and instead suggest that supposedly ‘morphological’ priming effects in L1 or L2 do not reflect processes of morphological decomposition, but are instead caused by prime-target similarity in form and meaning [2]. Finally, in a third group of studies, L1-L2 differences in morphological processing only occurred for some morphological phenomena, but not for others. For instance, Silva and Clahsen [17] tested masked priming effects with English derived and inflected forms in two groups of L2 speakers of English differing for their L1 (Chinese and German). Both groups showed significant morphological priming effects for derived forms, but no priming for inflected forms. Kırkıcı and Clahsen [18] observed a similar pattern in a masked priming study on L2 Turkish: Again, while L1 speakers showed similar priming effects for both derived and inflected Turkish forms, L2 learners of Turkish showed significant priming effects only for derived forms, with no priming for inflected words. Jacob, Heyer, and Veríssimo [19] also found an L2-specific difference between derivation and inflection in a masked priming study on native Russian L2 learners of German, with significant priming for both inflection and derivation in the L1 group, but priming only for derivation in the L2 group.

In a recent attempt to explain why L2 masked priming studies have come to different conclusions, Heyer and Clahsen [20] suggest that L1-L2 differences in morphological priming effects may, at least in some studies, be concealed because L2 speakers show seemingly morphological priming effects which are in fact not morphological in nature. Consider a prime-target pair such as *darkness-dark*: If *darkness* is morphologically decomposed, this should lead to the activation of *dark*, which would cause a morphological priming effect. However, *darkness* and *dark* are not only morphologically related, but also share a number of letters. Therefore, even if L2 speakers do not decompose a word such as *darkness*, they might nevertheless show significant priming effects for *darkness-dark*, because the two words are also related in orthographic surface form. Heyer and Clahsen [20] tested the processing of derived forms in L1 and L2 speakers of English and compared morphological priming effects to orthographic priming effects. For morphologically related prime-target pairs such as *darkness-dark*, they found significant priming effects in both groups. However, the L2 group, but not the L1 group, also showed significant priming effects for purely form-related prime-target pairs such as *scandal-scan*. The authors conclude that it is difficult to distinguish whether the observed morphological priming effect in L2 speakers is caused by morphological relatedness or by form similarity.

Indeed, the results from several studies investigating visual [21, 22] as well as auditory lexical processing [23–25] suggest that L2 speakers focus relatively more on surface form properties of words than L1 speakers, which, in priming studies, may lead to increased form priming effects. There is at least some evidence suggesting that such L2-specific form priming effects may even emerge when primes and targets are presented in different modalities. In a cross-modal priming experiment, Basnight-Brown, Chen, Hua, Kostić, and Feldman [26] investigated the L2 processing of irregular English past-tense forms with different degrees of form overlap to their stems: past-tense forms with nested stems (e.g. *drawn-draw*) versus forms with a stem change (e.g. *ran-run*). While L1 speakers showed similar priming effects for both types of irregular forms, the group of L1 Serbian-L2 English speakers showed larger priming effects for forms with nested stems. The authors explained this finding in terms of higher reliance on form level cues in L2 compared to L1 processing.

The presence of L2-specific form priming effects might explain why studies on L2 morphological processing have come to radically different conclusions: If L2 lexical processing focuses relatively more on surface form properties, this could cause form-based priming effects for morphologically related items in L2 speakers. As a result, both L1 and L2 speakers may show similar priming effects for pairs such as *walker*-*walk* (L1 speakers because of morphological similarity, L2 speakers due to surface form similarity), creating the illusion of similar underlying processing mechanisms in L1 and L2. Interestingly, as pointed out by Heyer and Clahsen [20], at least some of the above-mentioned studies that showed similar morphological priming effects for L1 and L2 speakers [2, 13] have also found significant surface form priming effects in their orthographic control conditions in the L2 group, but not in the L1 control group.

Significant orthographic masked-priming effects for L2 speakers have additionally been reported in two more recent masked morphological priming studies. M. Li, Jiang, and Gor [27] report the results from a study investigating the processing of English compounds in native English speakers and native Chinese L2 learners of English. The L1 group showed significant priming effects for both transparent and opaque compounds, but no priming for purely orthographically related items. The L2 group displayed similar priming effects for transparent and opaque compounds, but (unlike the L1 group) they also showed priming for orthographic word pairs with word-initial overlap. A similar pattern occurred in J. Li, Taft, and Xu’s [28] masked priming study on the L1 and L2 processing of English derived forms. Again, native English speakers displayed priming effects for morphologically related as well as pseudo-morphologically related word pairs, but no priming for orthographically related pairs, while native Chinese L2 learners of English showed priming effects in all three conditions.

## The present study

If it is indeed the case that L1-L2 differences in morphological processing are sometimes concealed because L2 speakers focus more on surface form properties of words, this raises the question why significant form priming effects only emerged in some, but not all masked priming studies on L2 morphological processing. In several of the studies discussed above, the included orthographic control conditions did not show any significant form priming effects for either L1 or L2 speakers. However, in some of them, as also mentioned by Heyer and Clahsen [20], the L2 groups showed at least some numerical trends for form priming effects [14, 17–19]. In sum, form priming effects in L2 speakers, while potentially highly relevant with regard to the issue of L2 morphological processing, seem to be quite unstable in masked priming.

At the same time, previous morphological processing studies have only discussed form priming effects in L2 specifically with regard to the masked priming paradigm. Specifically, Feldman and colleagues [2] suggest that masked priming may be unsuitable for studies with L2 speakers. This conclusion is based on the assumption that form priming effects are an artifact of masked priming and can easily be avoided by relying on experiments in which the primes are presented overtly (such as cross-modal priming): If the prime is only on screen for an extremely short time, L2 speakers might be unable to retrieve any morphological or semantic information contained in the stimulus. In other words, in order to be able to access anything more than just surface-form properties of a word, an L2 speaker may require a longer presentation time of the prime. It is possible, however, that the L2-specific orthographic effects are not specific to the masked priming paradigm, but instead reflect a general tendency of L2 speakers to focus more on orthographic surface form, as suggested by previous literature. In the latter case, form priming effects may also occur when the prime is presented overtly.

For the present experiment, we therefore decided to adopt an overt visual priming paradigm. In overt visual priming, unlike in masked priming, the prime is generally presented for around 200–300 ms and is thus clearly visible. This priming technique has reliably been employed in morphological processing research on L1, consistently showing priming effects for morphologically related word pairs [29, 30]. Some morphological processing studies on native speakers have suggested that bare orthographic similarity between prime and target may cause some facilitation at short prime durations (masked priming) [8, 31], while it consistently causes no facilitation or even inhibition at longer prime durations (overt priming) [29, 30, 32]. Furthermore, visual overt priming has been claimed to tap into later stages of processing in which word semantics come into play. Indeed, while masked priming has been found to at least considerably reduce semantic priming effects, semantic facilitation is robust under overt priming conditions [3, 32].

For the purposes of the present study, the overt visual priming technique offers at least two advantages. First, in visual overt priming, the clearly visible prime ensures that even L2 speakers have enough time to process it. This allows ruling out that any difference between the L1 and L2 group are caused by a difficulty of L2 speakers in dealing with short primes. Second, and more importantly, the inhibitory effects that have been reported for native speakers with purely orthographically related prime-target pairs (such as *scandal-scan*) [29, 30, 32] suggest that, if bare prime-target orthographic overlap has an effect on visual word recognition (in any direction), overt priming is more suitable to detect it than masked priming. If, as the literature seems to suggest, L2 speakers focus relatively more on the orthographic surface form of words during word recognition than L1 speakers [20–26], then they may show facilitation even at prime durations that would cause inhibition in an L1. This would strongly suggest that the larger reliance on orthographic cues in L2 morphological processing is a general characteristic of L2 processing, and it is not limited to the mechanisms that are tapped into by masked priming [2, 20].

In the present study, we determined L1 and L2 priming effects for morphologically related prime-target German word pairs, testing different types of morphologically complex words (derived and inflected), as well as priming effects for purely orthographically related pairs. For morphologically related word pairs, we expected that, under overt priming conditions, both L1 and L2 speakers would show significant priming effects. For purely orthographically related word pairs, in contrast, we expected to find different results for the two groups: Assuming that L2 speakers focus more on surface form properties during visual word recognition, and that this indeed conceals L1-L2 differences in the processing of complex words, we then expected the L2 group to display significant priming effects also for word pairs that are only related in orthography, while, in the L1 group, we expected inhibition. Based on Heyer and Clahsen [20], we assumed that morphological priming in L2 would be indistinguishable from orthographic priming. This would suggest that morphological priming effects in L2 may be in fact only accounted for by form-level overlap.

However, it is alternatively possible that the L2 form priming effects found in previous masked priming studies do not reflect a general, increased focus on surface form in L2 speakers, but are instead artifacts of the masked priming paradigm. If this is the case, form priming effects in L2 speakers should be specific to masked priming, and should not emerge in a paradigm in which subjects are given sufficient time to process the prime. This would lead to the conclusion that masked priming is an unsuitable paradigm to study morphological processing in L2 speakers, because of task-specific form artifacts in the L2 data [2].

It is important to keep in mind that visual overt priming has been shown to be more sensitive to semantic relatedness between primes and targets than masked priming. Indeed, prime-target pairs that are purely semantically related (e.g. *doctor-nurse*) typically also generate priming effects (unlike in masked priming) [30, 32]. Furthermore, word pairs such as *corner-corn*, which are semantically unrelated but superficially look as if they were morphologically related, generally show priming effects in masked priming, but not in overt priming [3, 30, 32, 33]. This is why the design additionally included a semantic control set. Based on previous morphological priming research with visual overt primes [30, 32], we expected to find semantic priming effects in both groups. This would suggest that, at this stage of processing, any morphological priming effect that we find might be at least partly semantic in nature. However, this would be true for both groups, and it would be therefore irrelevant for the main focus of the present study.

## Method

### Ethics statement

The study was approved by the ethic committee of the University of Potsdam (application number: 37/2011).

### Participants

Forty-eight advanced L2 learners of German (mean age 27.40, range 20–44, SD 5.01, 41 female) and forty L1 speakers of German (mean age 23.70, range 18–39, SD 3.80, 34 female) participated in the experiment in exchange for payment or course credits. All L2 participants were native speakers of Russian who acquired German later in life (mean age of acquisition 13.21, range 4–25, SD 5.48) and were living in Germany at the time of testing (mean age of arrival 20.02, range 7–36, SD 7.11). All participants were asked to fill out a short biographic questionnaire about their educational and linguistic background. The subjects in both groups had a similar level of education, varying from high-school to Master’s level, with one L2 subject additionally having a doctoral degree. All subjects of the L2 group indicated using German on a daily basis. L2 participants were additionally tested for their proficiency in German by means of the Goethe Institute Placement Test, a 30-item multiple-choice cloze test. The group achieved a mean score of 25.69 out of 30 (range 20–30, SD 2.63), which corresponds to a mean group proficiency of C1 (range B2-C2), labeled as ‘effective operational proficiency’ in the Common European Framework for Languages (CEFR). All subjects provided written informed consent for their participation.

### Materials

The design contained a morphological, an orthographic, and a semantic item set, allowing us to determine priming effects within each set and to subsequently compare these priming effects between the sets. All items were taken from the study by Jacob et al. [19]. Examples and details about relevant item features are shown in Table 1. Word-form and lemma frequency of all items were extracted from the webCELEX database (http://celex.mpi.nl/). A full list of stimuli is provided in the Appendix (see S1 Appendix).

**Table 1:** Mean item features (and standard deviations) and examples.

Primes			Targets		
WFF	LF	Length	WFF	LF	Length
Morphological set					
Inflected prime (*geändert* ‘changed’)					
22.1 (24.1)	82.6 (85.6)	7.9 (1.2)			
Derived prime (*Änderung* ‘change’)			Target (*ändern* ‘to change’)		
27.6 (36.3)	37.2 (47.9)	7.8 (1.2)	23.8 (21.7)	82.6 (85.6)	6.4 (1.0)
Unrelated prime (*klein* ‘small’)					
23.1 (21.3)	62.8 (107.6)	6.3 (1.0)			
Orthographic set					
Related prime (*Kasten* ‘box’)					
31.6 (48.3)	83.0 (144.2)	6.8 (1.7)	Target (*Kasse* ‘cash register’)		
Unrelated prime (*Schwan* ‘swan’)			36.3 (43.7)	71.5 (85.1)	5.5 (1.2)
32.0 (52.1)	91.2 (176.3)	6.5 (1.7)			
Semantic set					
Related prime (*Doktor* ‘doctor’)					
107.6 (217.5)	142.8 (252.9)	5.1 (1.2)	Target (*Arzt* ‘physician’)		
Unrelated prime (*Presse* ‘press’)			86.3 (170.4)	120.1 (205.0)	4.7 (1.2)
83.0 (140.2)	150.0 (240.7)	5.0 (1.3)			

WFF = Word form frequency per million, LF = lemma frequency per million, Length = number of letters.

All sets contained related, unrelated, and identity primes. In all of them, unrelated primes were matched as closely as possible not only to the related primes, but also to the identity primes, which allowed for additionally testing identity priming effects.

With regard to the morphological item set, note that several studies [17–19] suggest that L1-L2 differences in morphological processing may be specific to particular morphological phenomena such as inflected forms, and might not emerge for derived forms. To control for this, we measured morphological priming effects for both derived and inflected primes. The morphological set contained a total of 28 items. Within each of the 28 items, a German infinitive target verb (e.g. *ändern* ‘(to) change’) was combined with four different primes: (1) the verb’s past participle (e.g. *geändert* ‘changed’), (2) its corresponding derived -*ung* nominalization (e.g. *Änderung* ‘(the) change’), (3) an identity control prime (e.g. *ändern* ‘(to) change’), or (4) a matched unrelated control prime (e.g. *klein* ‘small’). Both the derived and inflected primes modified the verbal stem by adding three letters, thus affecting the overall length of the stem to the same extent. Note that, in *-ung* nominalizations, the affix *-ung* is added at the end of the stem, while, in past participles, a *ge-* is added at the beginning and a *-t* at the end of the stem. As a result, while the two conditions are matched with regard to the degree of *overall* orthographic overlap between prime and target (i.e. spelling changes to the stem were equated for inflections and derivations), they differ in the sense that the orthographic overlap is word-initial for derived and word-medial for inflected items. Inflected and derived primes were matched to each other for word-form frequency and length in letters. All unrelated control primes were semantically and orthographically unrelated to the target. Half of the unrelated primes were nouns and half were adjectives, matching the grammatical class of the related primes (note that German past participles can be used both as adjectives and verbs, but selecting verbs as unrelated primes was not possible because they would share the morphological ending -*en* with their targets). Unrelated and identity primes were matched for number of letters, lemma frequency, and word-form frequency, while all four prime types were matched for word-form and lemma frequency.

Because the stems of all targets in the morphological set were contained in their corresponding related primes, targets and related primes were not only morphologically, but also orthographically related. The orthographic set was thus designed to test for bare orthographic priming effects. The orthographic set included 24 morphologically simple targets. These were preceded by (1) an orthographically similar but otherwise unrelated prime word, (2) an identity control prime, or (3) an unrelated control prime. Related primes shared a series of letters with the targets, but without being semantically or morphologically related to them. In order to control for the fact that the orthographic overlap in the morphological items was word-initial for derived word pairs and word-medial for inflected pairs, and to check whether this has an effect on orthographic priming, half of the orthographically related prime-target pairs overlapped in the word-initial letters (e.g. *Wache*-*wachsen* ‘(the) guard-(to) grow’), similarly to those of the derived type in the morphological set, while the other half overlapped in their medial position (*Engel*-*Geld* ‘angel-money’), in the same way as in the inflected items. The amount of letter overlap in the morphologically (derived and inflected) and orthographically related prime-target pairs was calculated with Davis’ Match Calculator [34]. The related pairs in the two sets were comparable both in terms of absolute overlap (morphological set: mean 0.44, SD 0.40; orthographic set: mean 0.41, SD 0.38) and spatial coding (morphological set: mean 0.68, SD 0.13; orthographic set: mean 0.68, SD 0.11). Unrelated primes were morphologically simple nouns and verbs (e.g. *Schwan* ‘swan’), matched to the identity primes for word-form and lemma frequency and to the related primes for word class, length in letters, as well as word-form and lemma frequency.

Additionally, because the related prime-target pairs in the morphological set were also related in meaning, a semantic set was included to control for effects of semantic similarity. In the semantic control set, each target word was preceded by (1) a semantically related prime, (2) an identity prime, or (3) an unrelated control prime. The set included 24 semantically related noun-noun pairs, half of which were synonyms (e.g. *Doktor*-*Arzt* ‘doctor-physician’) and half associates (e.g. *Wolke*-*Himmel* ‘cloud-sky’), mimicking, respectively, the semantic relatedness between inflected and derived prime-target pairs. All related prime-target pairs were rated for sematic similarity by a group of 20 German native speakers using a 7-point Likert scale (1 = low semantic relatedness, 7 = high semantic relatedness), resulting in an average similarity of 5.19 (SD 0.85). Unrelated primes were morphologically simple nouns matching both the identity and the related primes for word class, number of letters as well as word-form and lemma frequency.

Targets in the orthographic and semantic sets were matched to those of the morphological set as closely as possible, but there were still substantial differences between them for both (word-form and lemma) frequency and length. Hence, following the suggestion of Sassenhagen and Alday [35], target properties were tested for inclusion as additional predictors in all the statistical models we fitted (see Data analysis for details). This way, the model outputs take into account differences in the targets from the different sets.

Four experimental lists were formed by means of a Latin Square design, each list containing only one of the prime-target pairs from each set, so that each participant saw each target only once. The morphological set contained four prime types (identity, inflected, derived, unrelated) and thus four different prime-target pairs. Hence, each of the four lists contained each target combined with one of the four prime types, so that each list presented a different prime-target combination. For the orthographic and semantic sets, because these contained only three prime types for each target (identity, related, unrelated) but the lists were four, one of three possible prime-target combinations occurred in two lists, but all lists contained the same distribution of prime types in both sets. From each list, we generated four additional lists containing the items in the reversed order, to counterbalance training or fatigue effects. This resulted in eight different presentation lists. The 76 experimental targets were mixed with 324 prime-target filler pairs in a pseudo-randomized order. All primes were existing words, while half of the targets were non-words, requiring a negative response in 50 per cent of trials. Non-words were obtained by changing one to three letters to existing morphologically simple and complex German words. Among filler targets and primes, simple words and morphologically derived adjectives, verbs, and nouns were equally distributed. Ten of the word filler pairs contained -*ung* nominalizations derived from *be-* prefixed verbs as primes and the corresponding simple stem as targets (e.g. *Benennung* ‘denomination’, from *benennen* ‘to denominate’- *nennen* ‘to name’). Overall, 18.69% prime-target pairs in the whole experiment consisted of related words, either because the prime and the target were morphologically, orthographically, or semantically related, or because they were identical.

### Procedure

Testing sessions took place in a quiet lab room. Subjects were tested on only one of the eight presentation lists and they were equally distributed across all lists. The experiment was run on the experimental software DMDX [36], which recorded reaction times (RTs) of participants in milliseconds. Participants were instructed that they would see a sequence of German words or non-words (target) in the center of the computer screen, each preceded by one distracting word (prime), and that they would have to decide as quickly as possible whether the target word was an existing word of German. Decisions were made by pressing two different buttons on a game-pad connected to the computer. Each trial started with a blank screen appearing for 500 ms. This was followed by a forward mask consisting of a series of hashes equal in length to the following prime, presented in the center of the screen for 500 ms. Next, the prime word was displayed for 200 ms, thus allowing for sufficient time to consciously perceive the prime for both groups of participants. The prime was immediately followed by the corresponding target word, which remained on the screen until the subjects performed their decisions or for a maximum of 500 ms. After the word had disappeared, the lexical decision could still be made for the next 4,500 ms. To ensure that the only difference from a masked priming experiment would be the longer exposure to the prime word, the procedure was exactly the same as in the masked priming experiment by Jacob et al. [19], except for the prime duration. This is why the experiment also included a forward mask although the prime was overtly presented.

### Data analysis

All subjects responded correctly to the majority of the experimental trials (accuracy range by participant in the two groups: L1 86.8%-100%; L2 77.6%-100%). Incorrect responses and timeouts were excluded from any further analysis (L1: 2.9%; L2: 5.2% of the data points). Based on visual inspection of the distribution of the response latencies, reaction times over 1,700 ms or below 300 ms were considered outliers and therefore removed from any subsequent analysis (L1: 0.1%; L2: 0.3% of the remaining data points).

Statistical analyses were performed on log-transformed RTs, in order to normalize RT distributions and reduce the influence of outliers [37]. The log-transformed RTs were analyzed with mixed-effect linear regression models, using the package lme4 [38] in the software ‘R’, version 3.3.2 [39]. We first tested priming effects in each stimulus set by fitting three separate mixed-effect models. All three models included the two fixed effects Group (L1, L2) and Prime Type (unrelated, inflected, derived, identity for the morphological set; unrelated, related, identity for the orthographic and semantic sets) and their interactions. The model testing for priming effects in the orthographic set additionally included the fixed factor Overlap Type (word-medial, word-initial) and its interactions with Group and Prime Type. For the fixed effects, contrasts were computed from the generalized inverse function [40], except for the factor Group, for which we used treatment contrast coding. With this combination of contrast coding, our models displayed effects of Prime Type (relatively to the baseline ‘unrelated’) only for the group selected as baseline: The baseline for Group was L1 and it was successively changed to L2, so that we could specifically assess whether all priming effects were significant in both groups of speakers. Furthermore, the models showed main effects of Group across different prime types, i.e. whether one group was faster than the other across all conditions. The model fitted on the orthographic set additionally provided effects of Prime Type (relatively to the baseline ‘unrelated’) across different overlap types, as well as effects of Overlap Type across different prime types, but still separately for the two groups.

We then further investigated priming in L2, with the aim of testing whether morphological priming was distinguishable from effects of orthographic relatedness. This analysis compared morphological priming effects both to orthographic and semantic priming, so that it reflected the full design of the study. Because the morphological set contained four levels but the two control sets contained three, we fitted two models, one comparing inflectional priming to priming in the two control sets, and the other comparing derivational priming to priming in the two control sets. For the orthographic set, we no longer distinguished between different types of overlap. Both models included the fixed factors Set (morphological, orthographic, semantic; baseline = morphological) and Prime Type (unrelated, related, identity; baseline = unrelated), together with their interactions. For these models, we were only interested in the relevant interactions between Set (morphological vs. orthographic or semantic) and Prime Type (unrelated vs. related). A significant interaction would hint at larger morphological than orthographic or semantic priming.

If it is the case that L2 speakers rely more on surface level properties, then this should also affect priming effects with identity primes, since, in identity prime-target pairs, all letters contained in the prime are also present in the target. Identity priming could thus be considered a case of particularly strong orthographic overlap. As a result, identity priming effects should be stronger in the L2 group than in the L1 group. To test for this, in a post-hoc analysis, we fitted a model testing identity priming in all three sets together, comparing the L2 group against the L1 group; this model included the fixed factors Group (L1, L2; baseline = L1) and Prime Type (unrelated, identity; baseline = unrelated), and their interaction. For this analysis, we were only interested in the interaction between Group and Prime Type: a significant interaction would signal larger identity priming in one of the groups.

For all models, parameters were estimated with restricted maximum likelihood. All models included random intercepts for subjects and targets. The initial model, only including the fixed effects for the experimental manipulations and the intercepts, was expanded step-wise with additional covariates and random slopes. We first tested for inclusion of the additional (centered) continuous covariates Length, Word-Form Frequency, and Lemma Frequency of the targets, as well as Trial Number, to account for differences in the target properties and for fatigue or habituation effects across trials. Once the fixed-effect structure was determined, we tested for inclusion of the relevant random slopes for each of the fixed effects that were part of the design. Both fixed effects and random slopes were tested for inclusion applying model comparisons. Each model containing one additional covariate or random slope was compared to the simpler model not containing it. We selected the model with the lower AIC (Akaike Information Criterion) score, if it significantly improved the model fit [41, 42], as tested with via likelihood ratio chi-square tests. The best-fit model for each analysis is reported in the Results section. For the purpose of model comparisons, parameters were estimated with maximum likelihood.

## Results

Table 2 shows means and standard deviations of raw RTs to targets for each experimental set and prime type, after outlier removal, as well as accuracy rates. The fact that both groups displayed very high accuracy rates across all conditions suggests that the experimental stimuli were familiar to them, and that they were fully capable to perform the task. In each set and in both groups, targets following identity primes were responded to more accurately and faster than those following unrelated primes, which shows that both groups were sensitive to the experimental design.

**Table 2:** Mean lexical decision times in milliseconds (and standard deviations), priming effects, and response accuracy.

Morphological set		unrelated	inflected	derived	identity
L1	RT	611 (154)	589 (156)	585 (150)	576 (175)
	Priming Effect	-	22	26	35
	Accuracy	95.4%	97.5%	97.1%	99.6%
L2	RT	733 (195)	697 (202)	683 (178)	652 (191)
	Priming Effect	-	36	50	81
	Accuracy	91.7%	96.7%	95.8%	97.9%
Orthographic set		unrelated	related		identity
L1	RT	582 (158)	590 (157)		540 (149)
	Priming Effect	-	-8		42
	Accuracy	94.7%	95.6%		98.1%
L2	RT	707 (208)	675 (192)		636 (216)
	Priming Effect	-	32		71
	Accuracy	92.2%	94.8%		96.9%
Semantic set		unrelated	related		identity
L1	RT	571 (132)	554 (132)		559 (177)
	Priming Effect	-	17		12
	Accuracy	96.6%	98.4%		98.4%
L2	RT	657 (167)	641 (152)		633 (209)
	Priming Effect	-	16		24
	Accuracy	92.2%	93.5%		96.6%

For the RT analyses, we first focused on the morphological set. Here, we expected to find significant priming in both participant groups. L1 speakers showed faster recognition of targets after inflected and derived primes compared to the unrelated baseline; the magnitude of priming for the inflected and derived primes was comparable (respectively, 22 and 26 ms). Similarly, L2 speakers also reacted faster following both inflected (36 ms) and derived primes (50 ms); however, responses were numerically faster after derived primes than after inflected primes (14 ms). Results from the linear-mixed effects model are shown in Table 3. First, we found a main effect of Group across all Prime Types (*t* = 4.324), indicating that the L1 Group was consistently faster than the L2 group in all conditions. The only significant interaction between Group (L2 vs. L1) and Prime Type was the one relative to the comparison between identity and unrelated Prime Type (*t* = -2.745), suggesting that repetition priming was larger for the L2 than the L1 group. By contrast, there was no significant interaction between Group and Prime Type (inflected or derived vs. unrelated) in the case of morphological priming, with either inflected or derived primes (both |*t*|s < 1.414). Importantly, priming effects with both inflected and derived primes were significant in both the L1 and L2 group (all |*t*|s > 2.471). By changing the baseline of Prime Type from ‘unrelated’ to ‘inflected’, the model provides priming effects for derived primes relative to inflected primes. The difference between derivational and inflectional priming was not significant for either of the two groups (L1: *b* = -0.0043, SE = 0.0153, *t* = -0.283; L2 *b* = -0.0155, SE = 0.0140, *t* = -1.102), and there was no significant interaction between Prime Type (derived vs. inflected) and Group (L1 vs. L2) (*b* = 0.0111, SE = 0.0207, *t* = 0.537), suggestive of no difference between L1 and L2 speakers for inflectional or derivational priming.

**Table 3:** Morphological set: Fixed effects from the linear-mixed effects model.

Fixed effects^a^	Estimate	Std. Error	t	Estimate	Std. Error	t
	Group baseline = L1			Group baseline = L2		
(Intercept)	6.3493	0.0268	236.493*	6.5078	0.0265	245.775*
Main Effect: Group (L2-L1), all Prime Types	0.1585	0.0367	4.324*			
Prime Type (identity-unrelated)	-0.0666	0.0153	-4.350*	-0.1239	0.0142	-8.733*
Prime Type (inflected-unrelated)	-0.0380	0.0154	-2.471*	-0.0565	0.0142	-3.970*
Prime Type (derived-unrelated)	-0.0423	0.0154	-2.750*	-0.0719	0.0143	-5.046*
Trial Number	-0.0297	0.0037	-8.113*			
WF Frequency	-0.0225	0.0085	-2.638*			
Length	0.0200	0.0085	2.337*			
Group (L2-L1) * Prime Type (identity-unrel.)	-0.0573	0.0209	-2.745*			
Group (L2-L1) * Prime Type (infl.-unrel.)	-0.0185	0.0209	-0.884			
Group (L2-L1) * Prime Type (derived-unrel.)	-0.0297	0.0210	-1.414			

^a^Formula of the best-fit model: log(RT) ~ Group * Prime Type + Trial Number + WF Frequency + Length + (1 | subject) + (1 + Group | target). Contrasts that are identical for both the L1 and L2 models are only reported for the L1 group.

We now turn to the results for the orthographic set. Here, unlike in the morphological set, we found differences between the L1 and L2 group. In the L2 group, mean RTs were faster when targets were preceded by an orthographically related prime as compared to the unrelated baseline (32 ms); on the contrary, the L1 group showed a numerical tendency towards inhibition (-8 ms). The linear-mixed effects model (Table 4) revealed a significant interaction between Group (L2 vs. L1) and Prime Type, for the contrast related versus unrelated Prime Type (*t* = -2.456). The effects of Prime Type (related vs. unrelated) in the two groups showed that the facilitation in the L2 group was significant (*t* = -2.222), while the numerical tendency for inhibition in the L1 group was not (*t* = 1.239). Note that we did not find any evidence of an effect of type of overlap (word-medial or word-initial) in either the L1 or L2 group, as there was no significant two-way interaction between Overlap Type (medial vs. initial) and Prime Type (related vs. unrelated) in either L1 or L2 (both |*t*|s < 1.078) and no significant three-way interaction between Group (L2 vs. L1), Overlap Type (medial vs. initial), and Prime Type (related vs. unrelated) (*t* = 1.264). Like in the morphological set, the model additionally showed a significant main effect of Group (*t* = 4.707), again because L1 speakers were generally faster than L2 speakers in the performance of the task.

**Table 4:** Orthographic set: Fixed effects from the linear-mixed effects model.

Fixed effects^a^	Estimate	Std. Error	t	Estimate	Std. Error	t
	Group baseline = L1			Group baseline = L2		
(Intercept)	6.3179	0.0277	228.127*	6.4675	0.0258	250.665*
Group (L2-L1)—all Prime Types and Overlap Types	0.1568	0.0333	4.707*			
Overlap Type (medial-initial)—all Prime Types	-0.0053	0.0291	-0.183	0.0020	0.0287	0.069
Prime Type (identity-unrelated)—all Overlap Types	-0.0909	0.0160	-5.671*	-0.1236	0.0148	-8.326*
Prime Type (related-unrelated)—all Overlap Types	0.0200	0.0161	1.239	-0.0332	0.0149	-2.222*
Trial Number	-0.0125	0.0044	-2.874*			
Length	0.0421	0.0138	3.060*			
Group (L2-L1) * Overlap T. (medial-initial)—all Prime Types	0.0073	0.0176	0.415			
Group (L2-L1) * Prime T. (identity-unr.)—all Overlap Types	-0.0327	0.0215	-1.521			
Group (L2-L1) * Prime T. (related-unr.)—all Overlap Types	-0.0532	0.0217	-2.456*			
Overlap T. (medial-initial) * Prime T. (identity-unrelated)	-0.0048	0.0350	-0.137	-0.0189	0.0322	-0.587
Overlap T. (medial-initial) * Prime T. (related-unrelated)	-0.0380	0.0352	-1.078	0.0213	0.0323	0.661
Group (L2-L1) * Overlap T. (med.-init.)* Prime T. (id.-unr.)	-0.0141	0.0467	-0.302			
Group (L2-L1) * Overlap T. (med.-init.)* Prime T. (rel.-unr.)	0.0593	0.0469	1.264			

^a^Formula of the best-fit model: log(RT) ~ Group *Overlap Type * Prime Type + Trial Number + Length + (1 | subject) + (1 | target). Contrasts that are identical for both the L1 and L2 models are only reported for the L1 group.

In the semantic control set, both L1 and L2 speakers showed facilitation, reacting faster to targets when these were preceded by primes related in meaning than by unrelated primes (respectively, 17 ms and 16 ms). Results from the best-fit model testing semantic priming are provided in Table 5. The interaction between Group (L2 vs. L1) and Prime Type (related vs. unrelated) was not significant (*t* = 0.042), suggesting no evidence for a difference in semantic priming for the two groups. The semantic priming effect was significant for both groups (both |*t*|s > 2.396). Again, L1 speakers were significantly faster than L2 speakers across all prime types (main effect of Group, *t* = 4.214).

**Table 5:** Semantic control set: Fixed effects from the linear-mixed effects model.

Fixed effects^a^	Estimate	Std. Error	t	Estimate	Std. Error	t
	Group baseline = L1			Group baseline = L2		
(Intercept)	6.3015	0.0261	241.502*	6.4439	0.0278	232.073*
Main Effect: Group (L2-L1), all Prime Types	0.1424	0.0338	4.214*			
Prime Type (identity-unrelated)	-0.0491	0.0140	-3.498*	-0.0793	0.0132	-5.988*
Prime Type (related-unrelated)	-0.0336	0.0140	-2.396*	-0.0327	0.0133	-2.459*
Trial Number	-0.0260	0.0039	-6.716*			
Length	0.0285	0.0118	2.427*			
Group (L2-L1) * Prime Type (identity-unrelated)	-0.0301	0.0191	-1.575			
Group (L2-L1) * Prime Type (related-unrelated)	0.0008	0.0192	0.042			

^a^Formula of the best-fit model: log(RT) ~ Group * Prime Type + Trial Number + Length + (1 | subject) + (1 + Group | target). Contrasts that are identical for both the L1 and L2 models are only reported for the L1 group.

As for identity priming, this was numerically larger for the L2 group in all sets (morphological set: L1 35 ms, L2 81 ms; orthographic set: L1 42 ms, L2 71 ms; semantic set: L1 12 ms, L2 24 ms). The best-fit model testing for differences in the magnitude of identity priming in the two groups included the fixed factors Prime Type (identity, unrelated) and Group (L2 vs. L1), their interactions, as well as the additional predictors Trial Number, Lemma Frequency and Length of the targets, and random slopes for Prime Type by subject and for Group by Target. The interaction between Group and Prime Type was found to be significant (*b* = -0.0406, SE = 0.0165, *t* = -2.459), confirming larger identity priming for L2 speakers.

Our results for the orthographic set show that orthographic priming effects in L2 speakers can also be found under overt priming conditions. What remains unclear is whether the facilitation found for both derived and inflected primes in the L2 group can be entirely explained in terms of orthographic overlap between prime and target, or whether the effect is, at least partly, morphological in nature. A superficial comparison between the numerical magnitudes of priming for morphological items in L2 (50 ms for derived and 36 ms for inflected items) with the priming effect for orthographically related primes (32 ms) suggests that derivational priming may potentially be stronger than orthographic priming while inflectional priming may be indistinguishable from orthographic priming. In order to test this, we fitted two additional models comparing priming with orthographically and semantically related primes to morphological priming after, respectively, inflected and derived primes. In these models, a significant interaction between Set (orthographic or semantic vs. morphological) and Prime Type (related vs. unrelated) indicates morphological priming effects beyond orthographic or semantic priming. For both comparisons, the best-fit models contained the additional predictors Trial Number and Length and Lemma Frequency of the targets, and no random slopes. In the model comparing priming after inflected primes to priming after orthographically and semantically related primes, the interactions between Set (orthographic vs. morphological; semantic vs. morphological) and Prime Type (related vs. unrelated) were not significant (respectively: *b* = 0.0280, SE = 0.0209, *t* = 1.340; *b* = 0.0227, SE = 0.0210, *t* = 1.083). In contrast, in the model comparing derivational to orthographic and semantic priming, the interaction between Set and Prime Type was significant for the comparison between morphological and orthographic priming (*b* = 0.0443, SE = 0.0208, *t* = 2.139), while it was only marginally significant for the comparison between morphological and semantic priming (*b* = 0.0393, SE = 0.0208, *t* = 1.891). In sum, the facilitation on target recognition we found in L2 for inflected primes was indistinguishable from an effect of bare orthographic and semantic relatedness between prime and target. Instead, L2 priming effects for derived primes were larger than priming resulting from bare orthographic overlap, while they were only marginally significantly larger than semantic priming effects.

## Discussion

The purpose of our study was to investigate the role of orthography in the L2 processing of morphologically complex words. A series of recent masked priming studies have shown L2-specific priming effects for word pairs that were orthographically, but not morphologically or semantically related [2, 13, 20, 27, 28]. However, other studies did not find any significant effect for orthographic priming in L2 [14, 18, 19]. In the present study, we aimed at clarifying the relative contribution of orthographic and morphological cues during visual word recognition in L2 processing. Because significant orthographic priming effects in L2 speakers have been reported in several, but not all masked priming experiments, we opted for an overt presentation of the prime, i.e. overt visual priming, which generally causes inhibitory effects for purely orthographically related prime-target pairs in L1 speakers and is therefore potentially more sensitive to modality-specific form-level effects. We investigated whether L2 orthographic priming effects occur even under overt visual priming conditions and whether morphological priming effects in L2 are distinguishable from effects of pure form-level overlap.

For morphologically related word pairs, our results showed significant priming effects in both the L1 and the L2 group, for both inflected and derived primes, thus confirming our expectations. By looking at the results from the morphological set in isolation, we might have concluded that L1 and L2 speakers do not differ in how they process morphologically complex words. However, the picture changes when we look at the items from the matched orthographic set. Here, we found significant form priming effects for the L2 group, but no priming for the L1 group, which suggests that the priming effects for morphologically related items in the L2 group might not be entirely morphological (or morpho-semantic, as it is the case in visual overt priming) in nature, but at least modulated by form similarity between prime and target. Note that the position of the orthographic overlap between prime and target (word-medial or word-initial) was not found to modulate the orthographic priming effect. This is also reflected in the morphological set where, if orthographic overlap position played a role, we should have observed a difference in the priming effect with inflected and derived primes, since the former share letters in word-medial position with their respective targets, while the latter overlap with their targets word-initially. Morphological and orthographic effects aside, we also observed significant facilitation for related word pairs in the semantic set, in both the native and non-native group. This is in line with previous research on overt visual priming on L1, which has shown that an overt presentation of the prime makes its semantic information available [32].

Additional evidence for surface form-level processing in the L2 group was provided by the significantly larger magnitudes of repetition priming for this group as compared to the L1 speakers. While we believe that identity priming can be informative of the mechanisms underlying L1 and L2 visual word recognition, we must acknowledge that identity prime-target pairs represent a case of full overlap not only in terms of orthography, but also in terms of semantics. Considering, however, that the two groups of speakers were found to differ with regard to orthographic, but not semantic priming, we believe that the larger identity priming effect in L2 is more likely to arise from orthographic cues. It is additionally also true that L2 speakers were slower than L1 speakers in their responses to targets following unrelated primes (morphological set: L1 611 ms, L2 733 ms; orthographic set: L1 582 ms, L2 707 ms; semantic set: L1 571 ms, L2 657 ms), which may leave more room for larger priming effects to emerge. However, L2 speakers were consistently slower than L1 speakers in all primed conditions, including responses to targets following identity primes (morphological set: L1 576 ms, L2 652 ms; orthographic set: L1 540 ms, L2 636 ms; semantic set: L1 559 ms, L2 633 ms).

A recent account by Grainger and Beyersmann [43] has suggested that morphological priming effects are the result of stem spotting mechanisms, which operate irrespective of whether the words are truly morphologically complex (i.e. even in pairs such as *scandal-scan*) and would therefore be orthographic in nature. According to this account, significant priming effects are observable with pairs such as *scanner-scan* but not with *scandal*-*scan* because, in the latter case, inhibition between prime and target due to their competing semantics would counterbalance a facilitatory priming effect. Consequently, this account would not explain the orthographic priming effects in L2 as the result of more enhanced focus on orthographic cues compared to native speakers, but rather as a result of less efficient use of semantic information, which would lead to lack of inhibition from competing semantics. However, if the L2 orthographic priming effect originates from weaker semantic representations (leading to a lack of semantic inhibition), this should have consequences for all experimental sets. Specifically, it should affect the strength of the priming effect in the semantic control set, which should be weaker in L2. However, L1 and L2 speakers showed semantic priming effects of similar magnitude. Because it is unlikely that the supposed weaker semantic representations of L2 speakers have an effect on the orthographic set, but not on the semantic set, we do not think that this account is compatible with our data. Furthermore, this account cannot explain the results from the cross-modal priming study by Basnight-Brown et al. [26], which showed an L2-specific effect of form overlap in morphologically related prime-target pairs (larger priming for pairs with larger overlap), hence in prime-target pairs that do not have competing semantics. We nevertheless concede that more research is needed to understand the exact mechanisms underlying orthographic priming effects and what regulates the occurrence of inhibitory versus facilitatory effects with purely orthographically related prime-target pairs.

Another relevant aspect of the Grainger and Beyersmann’s [43] account is that they explicitly speak of *edge-aligned* stem spotting, i.e. identification of stems that are either embedded word-initially or word-finally. In the present study, we tested morphological priming with complex words that overlapped with their targets not only word-initially, but also in word-medial position, and found that there was no difference in the priming effect for L2 speakers between the two different types of overlap. While word-medial overlap was not considered in the original account by Grainger and Beyersmann, our results suggest that this would be worth further investigating in order to update or improve the current models of morphological processing.

The present study extends the evidence for orthographic effects in L2 speakers from previous masked priming studies to overt visual priming. This suggests that the form priming effects typically observed in L2 speakers are not specific to the masked priming technique. Instead, the fact that L2-specific form priming effects persist even in overt visual priming, where L1 speakers normally show at least a trend for inhibition, suggests that, as claimed by Heyer and Clahsen [20], the larger reliance on (orthographic) surface form properties of complex words in L2 speakers is a general property of L2 visual word recognition, at least in lexical decision tasks. This is in line with previous L2 research from other domains [21–25].

If all the above is correct, this raises the question of why L1-L2 differences in morphological priming effects are in some studies concealed by orthography, but could be observed openly in other studies. Note that the studies mentioned above have investigated a wide range of different languages and morphological phenomena. It is conceivable that effects of surface form in L2 processing may be modulated by language-specific orthographic properties. For example, it has been claimed that orthography may be especially relevant for the processing of morphologically complex words in English, because English orthography is characterized by ‘morpho-orthographic spelling’ [44], i.e. how a word is spelled provides information about its morphological structure (see e.g. the morpheme -*ous* in *nervous*, whose phonological realization is equivalent to that of the non-morphemic ending <us> in *bonus* or <-ice> in *service*). Conversely, it has been observed that the abstract morphological structure of complex words in Semitic languages, consisting of root and pattern, forces their speakers to focus more on morphology than on surface-level properties of words as compared to Indo-European languages [45, 46]. As a consequence, non-native speakers of a given language may pay particularly attention to the cues that the language provides, and such enhanced attention to, in this case, orthographic cues may in turn result in orthographic priming effects. Clearly, more cross-linguistic research is needed to investigate to what extent orthographic overlap influences visual word processing in a non-native language across different languages and scripts.

When comparing the magnitude of priming for, respectively, derivation and inflection with orthographic priming in the L2 group, we found that the priming effect for derivation was significantly stronger than the orthographic priming effect, while the priming effects for inflected and orthographically related words were indistinguishable. This may suggest that, despite a degree of orthographic priming, L2 speakers may still be sensitive to the morphological structure of a complex word, at least when it comes to derived words. Consequently, although L2 speakers did not show any significant difference between derivational and inflectional priming in overt priming, inflected and derived words may still be processed differently, with inflectional priming being in fact only orthographic in nature and derivational priming being at least partly morphological. This would imply that the difference between inflection and derivation that has been described in the L2 masked priming literature [17–19, 47], with morphological priming effects for derivation but not for inflection, also applies, to a certain extent, to overt visual priming. As suggested by Jacob et al. [19], the L2 selective difficulty in dealing with inflectional morphology might be grounded in the properties of inflectional affixes, which are bare spell-outs of grammatical features and do not contain any semantic information. In contrast, derivational affixes, because of their semantic content, would be more salient and therefore easier to analyze. This may indeed particularly apply under overt priming conditions, were semantics plays a larger role than in masked priming. Note, however, that this suggestion of different processing mechanisms for inflected and derived words in L2 needs to be taken with some caution. First, this is the result of a comparison of morphological versus orthographic priming effects based on lexical-decision times for different item sets. Furthermore, if L2 processing of derived and inflected words is based on different mechanisms, this should lead to a difference in derivational versus inflectional priming effects as well: Priming with inflection would be significant, but still completely indistinguishable from orthographic overlap, while priming for derivation should be larger than both orthographic and inflectional priming. However, our analysis for the morphological items in the L2 group only showed a numerical tendency for larger derivational than inflectional priming. Finally, this interpretation implies that L2 speakers process derived and inflected words differently even under overt priming conditions, but the previous studies on L2 morphological processing that have reported differences in the L2 processing of inflection and derivation have explained this specifically with regard to the early, pre-lexical stage of processing addressed by masked priming. We are therefore reluctant to draw strong conclusions from this additional analysis and leave this open to further investigation. A study testing orthographic, inflectional, and derivational priming effects on the same targets, both under masked and overt priming conditions, may shed more light on this question. Furthermore, if it is true that inflectional, but not derivational priming in L2 is orthographic in nature, a study investigating both types of priming with different degrees of orthographic overlap to their targets should reveal that this modulates the magnitudes of inflectional, but not of derivational priming in L2, while it never modulates L1 priming effects. For German, for example, this would mean that fully embedded, highly overlapping, derived prime-target pairs such as *Apotheker*-*Apotheke* (‘pharmacist-pharmacy’) would yield equal priming effects to pairs with smaller overlap such as *Gärtner*-*Garten* (‘gardener-garden’), in both L1 and L2 speakers. Instead, irregularly inflected words without stem change (e.g. *geschlafen*-*schlafen* ‘slept-sleep’) should yield larger priming effects than irregularly inflected words with stem change (*geschlossen*-*schließen* ‘closed-close’) in L2, but equal priming magnitudes in L1. This would replicate the findings on inflection from the cross-modal experiment by Basnight-Brown et al. [26] with a visual priming task, and it would additionally extend their design to the domain of derivational morphology.

Although we realize that, under overt priming conditions, morphological priming effects may be at least partly semantic in nature (and this is indeed what our data would suggest), the focus of the present paper was rather on the aspects of visual word recognition of morphologically complex words that *differ* between L1 and L2 speakers. Even if the morphological priming effects we report in the present study were partly semantic, this would be the same for both groups of speakers, and hence of no relevance for the focus of the present study. We additionally note that, if semantics could explain all the morphological priming effects we report, then we would expect larger priming magnitudes for inflected than for derived primes, since inflection, unlike derivation, does not alter the meaning of the stem, but only spells out grammatical properties. However, this was clearly not the case in either of the two groups, which leads us to suggest that our experimental design was able to tap into a level of morphological processing that cannot be entirely restricted to the semantic similarity of complex words to their stems.

## Summary and conclusion

In sum, the most important finding from the current study is that, while L1 and L2 speakers displayed similar priming effects for morphologically related word pairs, L2 speakers additionally showed orthographic priming effects which did not occur in the L1 group. The fact that these L2-specific orthographic priming effects also emerged in overt visual priming suggests that they are not artifacts of a particular experimental paradigm (such as masked priming) which may be particularly challenging for this group, but may instead reflect a general tendency of L2 speakers to rely relatively more on orthographic surface form during visual word recognition than L1 speakers. This can cause seemingly morphological priming effects for morphologically related word pairs in L2 speakers, which are in reality not caused by the fact that prime and target share the same stem, but instead by similarity in orthographic surface form. As a consequence, if form similarity is not properly controlled for, direct comparisons of morphological priming effects for L1 and L2 speakers can lead to the wrong conclusions about L2 morphological processing, as the two groups might show similar priming effects for different reasons. In other words, the fact that L2 speakers focus relatively more on orthography may potentially conceal L1-L2 differences in morphological processing. This highlights the importance of including an orthographic control set in studies investigating morphological processing in L2, in order to be able to distinguish between morphological effects and effects caused by similarity in surface form.

In order to better understand the nature of the L2-specific orthographic priming effects and how this contributes to L2 morphological processing, future studies may test morphologically related prime-target pairs varying in the type and in the degree of orthographic overlap, similarly to the study by Basnight-Brown et al. [26] but with derived primes in addition to inflected primes, and under visual priming conditions. Testing orthographic, inflectional, and derivational priming on the same targets may also provide for an improved design to investigate which morphological processes are distinguishable from bare effects of form overlap. Moreover, future research may assess to what extent the enhanced focus on orthographic cues in the processing of complex words is truly a (task- and paradigm-independent) general property of L2 processing by employing other experimental tasks, such as production or semantic categorization tasks, but also if this additionally applies to phonological overlap, which may be assessed by means of auditory priming experiments. Finally, further research is needed to assess whether the effects we found are a function of other factors affecting L2 processing, like age of acquisition or proficiency.

## Supporting information

S1 Appendix Experimental items. Note that the letters *ä*, *ö*, *ü*, and *ß* are transcribed as, respectively, ‘ae’, ‘oe’, ‘ue’, and ‘ss’.
(CSV)

S1 Dataset Raw data with RTs and accuracy. (CSV)
